# Alien spiders in a palm house with the first report of parthenogenetic *Triaeris stenaspis* (Araneae: Oonopidae) infected by *Wolbachia* from new supergroup X

**DOI:** 10.1038/s41598-025-93540-1

**Published:** 2025-03-19

**Authors:** Paweł Szymkowiak, Edyta Konecka, Tomasz Rutkowski, Aleksandra Pecyna, Przemysław Szwajkowski

**Affiliations:** 1 Department of Animal Taxonomy and Ecology, Faculty of Biology, Adam Mickiewicz University Poznań, Uniwersytetu Poznańskiego 6, 61-614 Poznań, Poland; 2 Department of Microbiolgy, Faculty of Biology, Adam Mickiewicz University Poznań, Uniwersytetu Poznańskiego 6, 61-614 Poznań, Poland; 3 Department of General Zoology, Faculty of Biology, Adam Mickiewicz University Poznań, Uniwersytetu, Poznańskiego 6, 61-614 Poznań, Poland; 4 Poznań Palm House, Matejki 18, 60-767 Poznań, Poland

**Keywords:** Colonization, Endosymbionts, Invasive species, New species to Poland, Novel bacterial phylogeny lineage, Urban ecological island, Invasive species, Symbiosis, Bacterial genes, Animal migration, Biodiversity, Invasive species, Urban ecology, Phylogenetics

## Abstract

Palm houses in Europe serve as urban biodiversity hot spots for alien spiders. As a result of several years of research in the Poznań Palm House, we documented the occurrence of 14 spider species, 9 of which were alien to Europe: *Coleosoma floridanum*, *Hasarius adansoni*, *Howaia mogera*, *Ostearius melanopygius*, *Parasteatoda tabulata*, *Parasteatoda tepidariorum*, *Scytodes fusca*, *Spermophora kerinci* and *Triaeris stenaspis*. The most abundant species was *C. floridanum* (39.9%). Three spider species were recorded for the first time in Poland: *C. floridanum*, *S. fusca* and *S. kerinci*. We studied the occurrence of endosymbiotic *Wolbachia* and *Cardinium* in parthenogenetic *T. stenaspis* and recorded for the first time the occurrence of *Wolbachia* in this spider. The endosymbiont was characterized based on the sequences of six bacterial housekeeping genes: 16S rRNA, *coxA*, *fbpA*, *ftsZ*, *gatB* and *hcpA*. Our phylogenetic reconstruction of *Wolbachia* supergroups revealed that the bacteria recovered from the spider formed distinct lineages in relation to all known supergroups. We assigned it to a novel supergroup X with unique sequences within the 16S rRNA and *ftsZ* genes. We discussed faunistic results in terms of long-term survival rates and the risk of invasion of alien species of spiders.

**Supplementary Information:**

The online version contains supplementary material available at 10.1038/s41598-025-93540-1.

## Introduction

Spiders disperse actively on land through their own locomotor abilities, passively in the air through air currents (ballooning)^1,2^ and with human involvement (anthropodispersal)^3^. They are among the first colonizers capable of finding suitable niches for settlement in a foreign environment. This ability makes introduced species potential invaders to local faunas^2–9^. Over the last 200 years, many alien species have been recorded in Europe, and some of them have become established^3,8,10,11^. They often arrive in Europe with global trade products^10^, predominantly with imported fruits and plants^11^. Spiders of tropical origin introduced to Europe, due to their preferences for high temperatures and humidity, usually find suitable places to settle and develop stable populations in palm houses and botanical gardens^7,12–19^. Such locations offer similar living conditions to those in the subtropics and tropics, turning them into unique urban ecological islands and hot spots of biodiversity for alien invaders^20^.

Studies on alien invertebrates in the Poznań Palm House have been conducted extensively in the past^20–26^. Several alien species of arachnids have been documented, including those belonging to Acari^27–31^ and Schizomida^32^. Initial research on spiders was conducted by Woźniczko^33^ as part of her master’s thesis, but the results were never officially presented except the information on the occurrence of the jumping spider *Hasarius adansoni* (Audouin, 1826)^34,35^.

Colonization of distant habitats far from the parental distribution range is much more intense and successful when invaders exhibit a parthenogenetic type of reproduction and have high ecological tolerance^36–39^. Parthenogenesis can be induced by bacterial endosymbionts, such as *Wolbachia* (phylum Pseudomonadota, class Alfaproteobacteria, order Rickettsiales, family Ehrlichiaceae) and *Cardinium* (phylum Bacteroidota, class Sphingobacteriia, order Sphingobacteriales, not assigned to family). *Wolbachia* is considered one of the most common bacterial endosymbiont among arthropods^40,41^. The associations between the microorganism and the host can take various forms, including changes induced by *Wolbachia* in the proportion of females and males in the invertebrate population. *Wolbachia* can induce several reproductive phenotypes in their hosts, including feminization, resulting in death of embryonic males^42^, or the conversion of genetic males into functional phenotypic females^43^. The bacteria can also lead to cytoplasmic incompatibility, which occurs after mating between males infected with certain bacterial strains and females either uninfected or infected with an incompatible bacterial strain. The consequence is embryonic death^44^. *Wolbachia* may also be the causative agent of parthenogenesis^41,45^ in host species by modifying mitosis or meiosis^46^.

On the basis of the phylogeny of housekeeping genes or whole-genome typing methods, the genus *Wolbachia* has been divided into 21 supergroups (A-W). An examplary set of genes comprises 16S rRNA, *coxA*—coding for cytochrome c oxidase, *gatB*—coding for glutamyl-tRNA(Gln) amidotransferase, *hcpA—*coding for conserved hypothetical protein, *ftsZ—*coding for prokaryotic cell division protein and *fbpA—*coding for fructose-bisphosphate aldolase^47–49^.

Although reproduction by parthenogenesis could offer advantages for the colonization of new environments by introduced species, it is a rare phenomenon in spiders. In most cases, it has been discussed speculatively rather than confirmed with results^50–56^. The only reliable studies were performed by Korenko et al.^36^, who studied a population of *Triaeris stenaspis* Simon, 1892, and Lake^57^, who maintained an immature female of the huntsman spider *Isopoda insignis* Simon, 1897 (currently included in the genus *Holconia*^58^). Both studies were conducted under laboratory conditions. It is known that no males of *T*. *stenaspis* have been collected together with females^36,59^. Although *T. stenaspis* reproduces by thelytokous parthenogenesis, the occurrence of endosymbiotic bacteria like *Wolbachia* or *Cardinium*, which could manipulate the sex ratio towards a lack of males and induce parthenogenesis, has not been confirmed^36^. This species is found in some tropical and subtropical areas of the world^59^ and has been introduced into Europe with cultivated pot plants^60^. Its presence has been observed exclusively in palm houses^11,61^. *T. stenaspis* has so far been reported from the aforementioned locations in Austria^62^, Czech Republic^61^, Finland^15^, Germany^7,16^, Hungary^63^, Ireland^64^, the Netherlands^65^, Poland^60,66^, Slovakia^67,68^, Switzerland^65,69^ and United Kingdom^64^. Some sites of occurrence (Danmark, France) were obtained from national checklists of spiders published online^70,71^.

The primary objective of our study was to analyze the spider species composition, including the proportion of alien species inhabiting the Poznań Palm House. Additionally, we aimed to study the occurrence of *Wolbachia* and its genetic variability in *T. stenaspis*, as it may manipulate the spider’s reproduction towards parthenogenesis as an adaptation for survival in isolated conditions. Furthermore, we performed a phylogenetic analysis of *Wolbachia* to explore its relationships with other endosymbiont strains belonging to existing supergroups.

## Materials and methods

### Study sites

The Poznań Palm House, established in 1911, serves as an educational and exhibition facility. It is the largest palm house in Poland and one of the largest in Europe. Comprising 12 pavilions, the facility showcases vegetation representative of subtropical climate (pavilion I), temperate climate (pavilions II and III), succulents of America (pavilion IV), vegetation of the tropics (pavilions V and VI), aquatic vegetation (pavilion VII), tropical forest undergrowth (pavilion VIII) and xerophytic vegetation and savannas (pavilion IX)^72^.

### Study material

Spiders were collected in 2013 (29/10 and 25/11), 2014 (17/11), 2015 (02/03, 20/04, 02/11, 17/11 and 14/12), 2016 (06/04), 2023 (13/03, 20/03 and 17/04). The collection sites included pavilions I, II, V, VI, VII, IX, as well as the breeding room and underground corridors with the heating system of the palm house. The species composition of spiders in the Poznań Palm House, indicating abundance and occupied habitats, is presented in Table 1. Additional data on the sampled material and collectors is shown in Table S1, available in the online Supplementary Material.

**Table 1:** Composition of spider species in Poznań Palm House with notes on abundance and occupied habitat.

Species	Habitat	Male	Female	Juv.	Total
*Amaurobius ferox*	Crevice, wall	4	2	4	10
*Amaurobius* sp.	Crevice, wall			1	1
*Araneus diadematus*	Branches of trees, bushes		1		1
Coleosoma floridanum*	Branches of trees, dry bamboo leaves on ground, litter	19	48	22	89
*Dysdera* sp.	Litter			1	1
Hasarius adansoni*	Varied habitats	5	1	3	9
Howaia mogera*	Dry bamboo leaves on ground	6	15	10	31
Ostearius melanopygius*	Litter	3	8	4	15
Parasteatoda tabulata*	Branches of trees, dry bamboo leaves on ground	1	1	12	14
Parasteatoda tepidariorum*	Underground wall	0	1	0	1
*Pholcus opilionoides*	Underground wall	2	1	1	4
*Pirata* sp.	Litter			1	1
Scytodes fusca*	Dry bamboo leaves on ground, underground tunell wall	5	11	12	28
Spermophora kerinci*	Dry bamboo leaves on ground	0	2	1	3
*Steatoda grossa*	Underground wall	0	0	2	2
*Tegenaria domestica*	Underground wall, under the flower pot, under the box	0	2	6	8
*Tegenaria* sp.	Underground wall			1	1
Triaeris stenaspis*	Dry bamboo leaves on ground, underground wall, litter	0	4		4

*Alien species.

### Study techniques

The collection process involved hand sorting from beneath stones, logs of trees, crevices, walls and window panes. Additionally, dry bamboo leaves and other litter material from the ground were sieved.

Species identification was carried out using an Olympus SZX12 stereomicroscope, and specimens were photographed using an Artcam 500MI digital camera. Images were processed using Quick Photo Camera 2.3 software and Adobe Photoshop CS2. The reproductive organs of females of the species *Coelosoma. floridanum *Banks, 1900, *Pholcus* spp. and *Howaia mogera* (Yaginuma, 1972) were dissected and cleared with lactic acid. All specimens were stored in 75% ethanol.

### Genetic analysis of *Triaeris stenaspis*

Total DNA from the legs of one spider specimen was extracted using the Genomic Mini kit (A&A Biotechnology) for universal genomic DNA isolation according to the manufacturer’s recommendation. The COI gene was detected by PCR amplification with LEPF1 and LEPR1 primers, whose sequences and annealing temperature are described in Hebert et al.^73^. PCR reactions were carried out in mixtures containing: 3 µl DNA, 1.5 µl 10× DreamTaq Buffer (Thermo Scientific), 0.3 µl 10 mM dNTP (A&A Biotechnology), 0.75 µM each primer (Oligo.pl), 0.4 U DreamTaq DNA Polymerase (Thermo Scientific) and sterile bidistilled water to a final volume of 15 µl. Each reaction contained a sample without template DNA, which served as a negative control. PCR products were electrophoresed in an agarose gel (NOVA Mini, Novazym) and sequenced with BigDye Terminator v3.1 using an ABI Prism 3130XL Analyzer (Applied Biosystems).

### Identification of *Wolbachia* and *Cardinium* endosymbionts in *Triearis stenaspis*

Detection of *Wolbachia* and *Cardinium* endosymbionts^68^ was performed for four spider specimens. DNA isolation from individual specimens was performed as described above. Endosymbiont genes were detected by PCR amplification using primers whose sequences and annealing temperatures were reported by Brown et al.^69^ and Zchori-Fein & Perlman^70^. PCR reactions, gel electrophoresis in agarose gel and sequencing was carried out as described above. The BLASTn algorithm was used to compare DNA sequences with data deposited in GenBank.

### Molecular analysis of *Wolbachia* genes in *Triaeris stenaspis*

Molecular characterization of *Wolbachia* was based on the analysis of the following housekeeping gene sequences: 16S rRNA^69,74^, *coxA*, *fbpA*, *gatB*, *hcpA*^47^ and *ftsZ*^75^. The primer sequences and annealing temperatures are presented in Table S2.

Amplifications were performed in a 15 µl mixtures as described above. The PCR cycling profile was as follows: 95 °C for 5 min; 40 cycles at 95 °C for 30 s, annealing (Table S2) for 30 s, and 72 °C for 45 s, and a final elongation at 72 °C for 5 min. Subsequently, the amplified products were subjected to electrophoresis, sequencing and BLASTn analysis as described above. The sequences of the 16 S rRNA, *coxA*, *fbpA*, *ftsZ*, *gatB* and *hcpA* genes derived from *Wolbachia* inhabiting *T*. *stenaspis* have been deposited in GenBank.

The 16S rRNA sequence of *Wolbachia* from *T*. *stenaspis* was analyzed for nucleotide variations between bacterial strains from different *Wolbachia* supergroups, with specific focus on regions corresponding to individual positions in *Escherichia coli* 16S rRNA. Additionally, 16S rRNA was analyzed for the presence of a unique sequence specific to *Wolbachia* from the spider. The *ftsZ* gene sequence was also examined for the presence of a specific sequence characteristic for *Wolbachia* from *T*. *stenaspis*.

### Phylogeny of *Wolbachia* from *Triaeris stenaspis*

The sequences of the 16S rRNA, *coxA*, *fbpA*, *ftsZ*, *gatB* and *hcpA* genes of *Wolbachia* isolated from *T*. *stenaspis* were aligned with *Wolbachia* loci representing different phylogenetic supergroups (A–W) using MEGA 11 software^76^. NCBI accession numbers for sequences used in the phylogenetic analysis are presented in Figs. S1–S6 and Table S3 available in the online Supplementary Material. The alignments of the obtained sequences were constructed using ClustalW^77^. Single gene sequences were aligned, and then a multigene alignment was created using data of 33 *Wolbachia* strains from various hosts together with *T*. *stenaspis*. An outgroup of *Ehrlichia* spp. sequences was added to the analysis. The jModelTest 2 software^78,79^ was used to select the appropriate model of sequence evolution. The maximum likelihood bootstrap support was determined using 1000 bootstrap replicates. The HKY + G + I model was chosen for the 16S rRNA sequence data. For the *coxA* and *fbpA* genes, the HKY + G model was used. The GTR + G model was selected for the *gatB* and *hcpA* genes, as well as for concatenated sequence data of the six loci (16S rRNA, *coxA*, *fbpA*, *ftsZ*, *gatB* and *hcpA*). The TrN + G model was selected for the *ftsZ* sequence. Gene recombination between strains was detected by the φ test using SplitsTree4 software^80^.

Additional phylogenetic analysis was performed based on concatenated sequence data of the 16S rRNA, *coxA*, *fbpA*, *ftsZ*, *gatB* and *hcpA* genes of *Wolbachia* from *T*. *stenaspis* and *Wolbachia* supergroups A and B from other spider species using MEGA 11, ClustalW and jModeltest as described above. The GTR + G model was selected for this analysis. NCBI accession numbers for sequences used in the phylogenetic analysis are listed in Table S4 available in the online Supplementary Material.

## Results

### Composition of species

The material for analysis included 223 spider specimens collected between 2013 and 2023. Fourteen spider species were identified to the species level, including 9 belonging to species alien to Europe: *C. floridanum*, *H. adansoni*, *H. mogera*, *Ostearius melanopygius* (O. P.-Cambridge, 1879), *Parasteatoda tabulata* (Levi, 1980), *Parasteatoda tepidariorum* (C. L. Koch, 1841), *Scytodes fusca* Walckenaer, 1837, *Spermophora kerinci* Huber, 2005 and *T. stenaspis* (Table 1; Fig. 2a–o).

### Abundance of alien species

The most numerous species in our study was *C. floridanum*, constituting 39.9% of all spiders. This species was predominantly found at ground level, specifically in fallen, dry banana leaves. The proportion of the next four most abundant species was in the range of 6.3–13.9%: *H*. *mogera* (13.9%), *S. fusca* (12.6%), *O. melanopygius* (6.7%) and *P. tabulata* (6.3%) (Table 1).

### Sex ratio of alien species and sexual dimorphism in *C. floridanum*

**Fig. 1:**
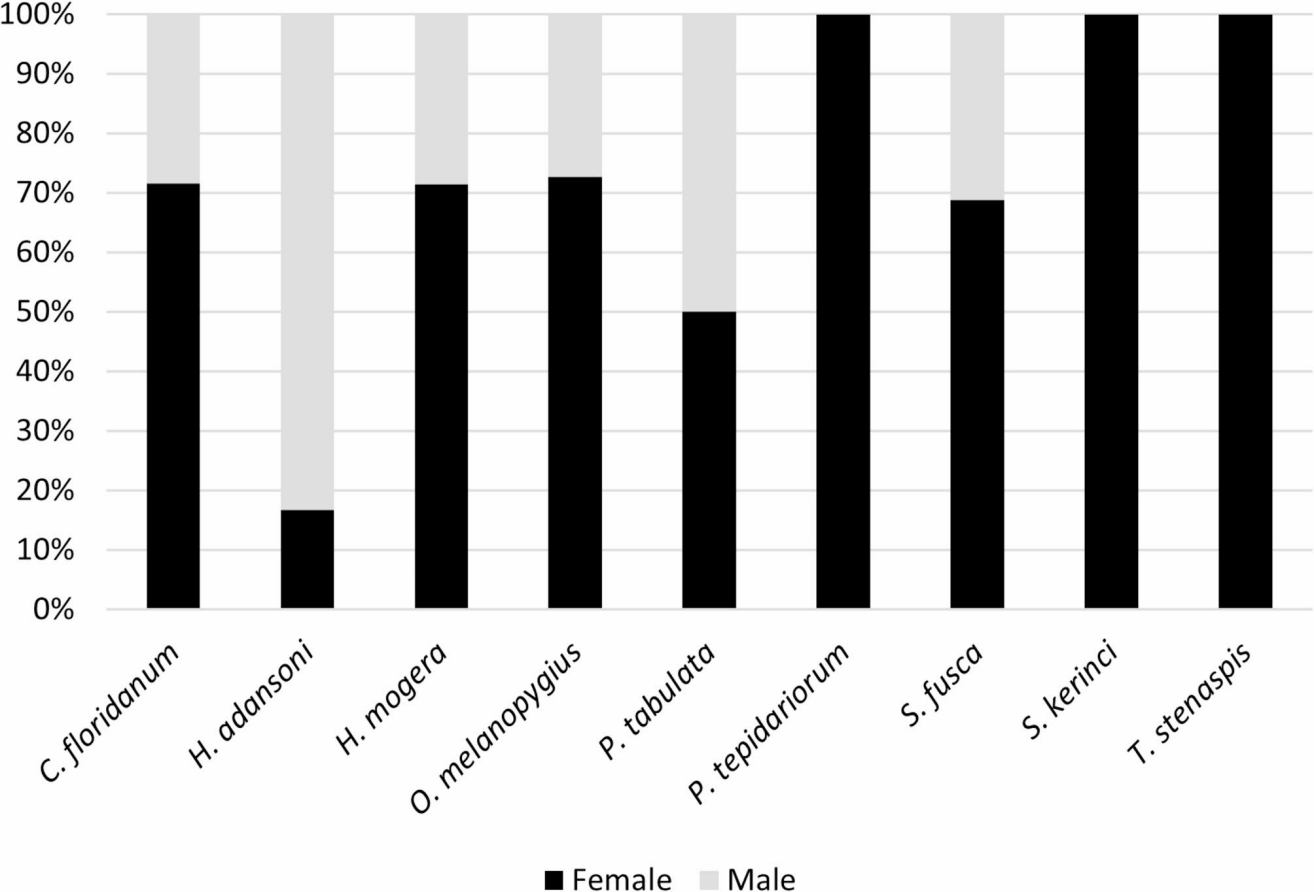
Sex ratio of alien species.

For *C. floridanum*, *H. mogera*, *O. melanopygius* and *S. fusca*, sex ratio was over 2, while for *H. adansoni*, it was 0.2. An equal sex ratio was observed for *P. tabulata*, while only females were recorded for *P. tepidariorum*, *S. kerinci* and *T. stenaspis* (Fig. 1). In the case of the latter species, only single specimens were recorded.

**Fig. 2:**
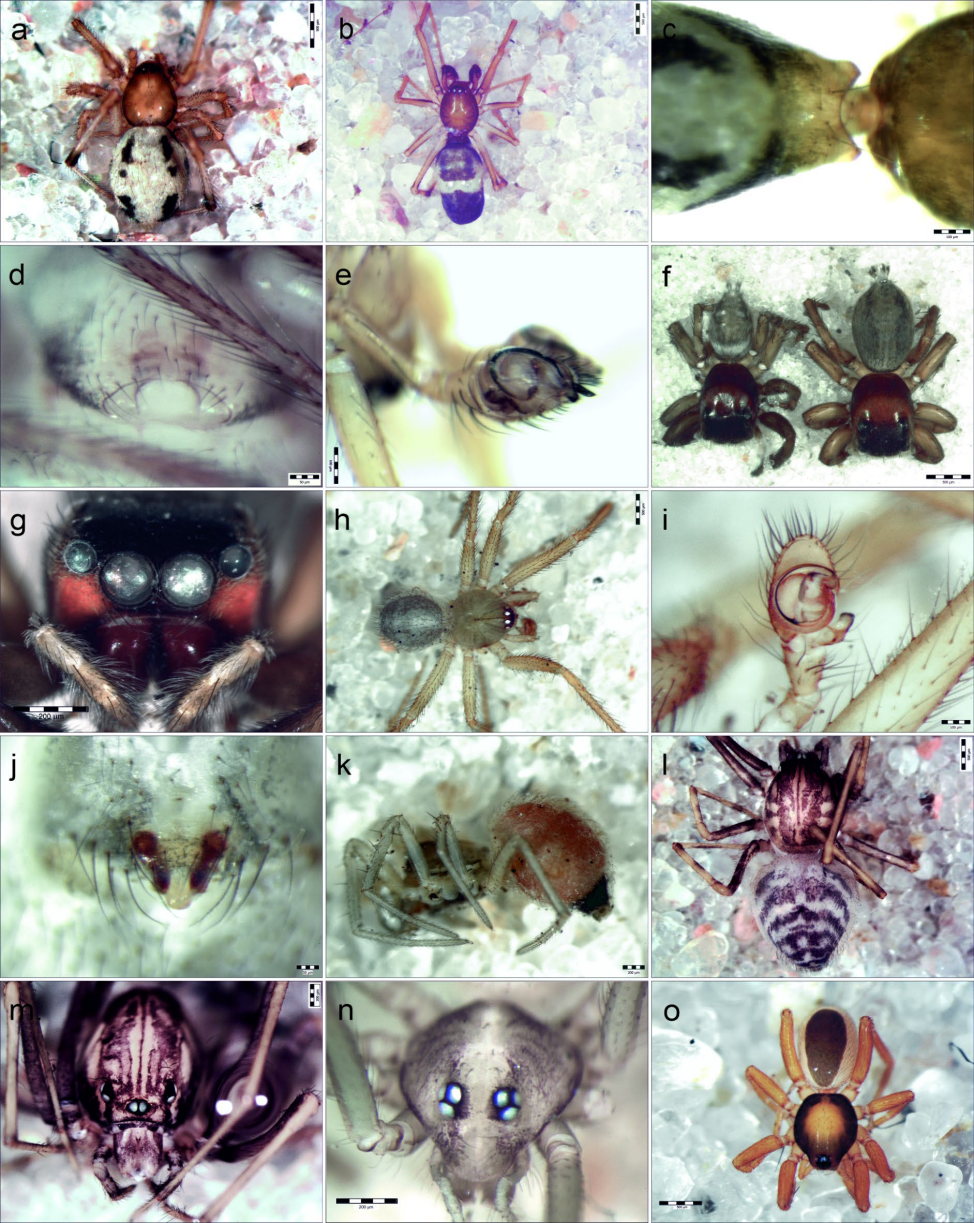
Alien spiders in the Palm House in Poznan, Poland. (**a**) *Coleosoma floridanum*, female, dorsal view, (**b**) *C. floridanum*, male, dorsal view, (**c**) *C. floridanum*, male sclerotized appendages located anteriorly on the abdomen, (**d**) *C. floridanum*, epigyne, (**e**) *C. floridanum* male palp, ventral view, (**f**) *Hasarius adansoni*, male and female, dorsal view, (**g**) *H*. *adansoni*, cephalothorax of female, frontal view, (**h**) *Howaia mogera*, male, dorsal view, (**i**) *H. mogera*, male palp, (**j**) *H*. *mogera*, epigyne, (**k**) *Ostearius melanopygius*, lateral view, (**l**) *Scytodes fusca*, female, dorsal view, (**m**) *S*. *fusca*, cephalothorax of female, frontal view, (**n**) *Spermophora kerinci*, cephalothorax of female, frontal view, (**o**) *Triaeris stenaspis*, female, dorsal view.

*Coleosoma floridanum* was characterized by the highest sexual dimorphism among the species studied. Females had a slightly oval, sometimes spherical abdomen with black spots running in two rows on the dorsum (Fig. 2a). Male abdomen was elongated and slightly narrow in 2/3 of its length, with a light transverse stripe on a dark background (Fig. 2b). The coloration may include a combination of dark and white spots. Additionally, males possessed characteristic two appendages on the sclerotized ring located anteriorly in the abdomen (Fig. 2c). The terminal apophysis of the male palp extended over the bulbus (Fig. 2e).

*Coleosoma floridanum*, *S. fusca* and *S. kerinci* were recorded for the first time in Poznań Palm House. They are new species in the fauna of Poland.

### Genetic analysis of *Triaeris stenaspis*

The 619-bp sequence of *T*. *stenaspis* COI gene has been deposited in GenBank under accession no. OR456447 and is an important source of molecular data for comparative studies. Comparative analysis showed the highest identity of 82.57% to Oonopidae sp. (accession no. OP242080) and 82.47% with Aranea sp. (accession no. OL694580). The sequence also exhibited identities of 80% and 80.78% with *T*. *stenaspis* (accession nos. KX536938 and KX536955, respectively).

### Identification of parthenogenesis-inducing bacterial endosymbionts in *Triaeris stenaspis*

For the first time, we have identified *Wolbachia* in the parthenogenetic spider species *T*. *stenaspis*. Three females out of four collected were infected. *Cardinum* was not found.

### Molecular characterization of *Wolbachia* from *Triaeris stenaspis*

We successfully amplified six *Wolbachia* housekeeping genes in *T*. *stenaspis*: 16S rRNA, *coxA*, *fbpA*, *ftsZ*, *gatB* and *hcpA*. All six genes were identified in three studied females of *T. stenaspis*. The obtained sequences were identical in studied specimens. *Wolbachia* genes originated from one female were used for phylogenetic analyses. NCBI accession numbers for the obtained *Wolbachia* sequences are as follow: OR457752 (16S rRNA), OR450018 (*coxA*), OR462179 (*fbpA*), OR462180 (*ftsZ*), OR462181 (*gatB*) and OR462182 (*hcpA*). The 443-bp region of the *Wolbachia* 16S rRNA sequence in *T*. *stenaspis* showed 99% identity to *Wolbachia* 16S rRNA from the isopteran insect *Nasutitermes takasagoensis* (Shiraki, 1911) deposited in GenBank under accession no. DQ837200. The amplicon of *coxA* (429 bp) showed the highest identity (93.56%) with bacterial sequences of the hemipteran insect *Nilaparvata muiri* (accession no. GU289807). The *fbpA* sequence of 436 bp showed the highest identity of 92.36% with the *Wolbachia* gene from the lepidopteran insect *Apotomis betuletana* (Haworth, 1811) (accession no. OX366320). The 330-bp sequence of the *ftsZ* gene of *Wolbachia* in *T*. *stenaspis* revealed the highest identity of 91.52% with the sequence of *Wolbachia* in the siphonapteran insects *Ctenocephalides felis* (Bouche, 1835) [accession nos. (1) CP051157—DNA sequence of *Wolbachia* strain *w*CfeJ, (2) CP116768—strain *w*CfeJ and (3) AJ628415—strain *w*Cfe]. The *gatB* sequence of 393 bp showed the highest identity (91.49%) with the endosymbiont gene from the hemipteran insect *Cystococcus echiniformis* Fuller, 1897 (accession no. MW511352). The PCR product of *hcpA* (469 bp) revealed the highest identity of 90.17% with the *Wolbachia* sequence from the odonate insect *Ischnura elegans* (Vander Linden, 1820) (accession no. OX366371).

The 16S rRNA sequences of *Wolbachia* from *T*. *stenaspis* and bacteria from other hosts representing supergroups A–W were compared based on nucleotide differences. In the gene of the spider-derived microorganism, we recorded a T nucleotide at position corresponding to position 747 in 16S rRNA of *E*. *coli*. Nucleotides A or C were found in the same position of *Wolbachia* 16S rRNA from supergroups A–W. Additionally, a unique 5’TCATATC-3’ sequence was found in the 16S rRNA gene of the endosymbiont from *T*. *stenaspis*. The sequence was located at positions 745–749 corresponding to the sequence of the 16S rRNA gene of *E*. *coli* (Fig. S7). We have also identified a unique 5’-CTTACAC-3’ sequence for *Wolbachia* from the spider in the *ftsZ* gene. The location of this sequence was determined at positions 612–616 in relation to *ftsZ* of *Wolbachia* from *Drosophila sturtevanti* Duda, 1927 (accession no. CP050531). An alignment showing the unique *ftsZ* sequence of *Wolbachia* supergroup A is illustrated in Fig. S8.

### *Wolbachia* phylogeny

**Fig. 3:**
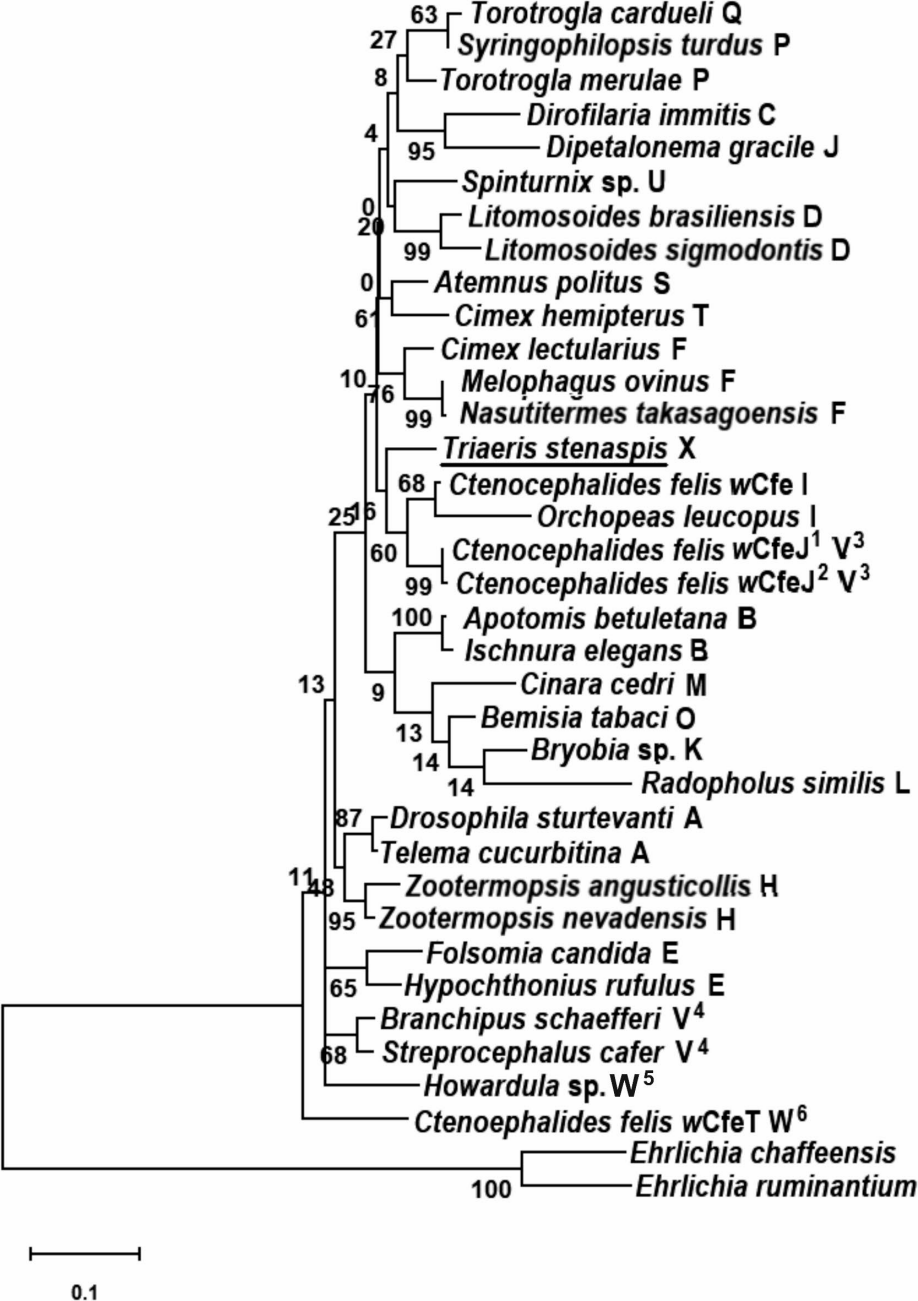
Maximum likelihood reconstruction of *Wolbachia* supergroup phylogeny based on concatenated sequence alignments of six bacterial loci (16S rRNA, *coxA*, *fbpA*, *ftsZ*, *gatB*, *hcpA*) using MEGA 11 software. Strains are denoted by their host names, except for outgroup bacteria. Capital letters indicate individual *Wolbachia* supergroups. Bar, substitutions per nucleotide. Bootstrap values based on 1000 replicates are shown on branches. ^1^complete genome of the strain *w*CfeJ deposited in GenBank under accession no. CP051157. ^2^complete genome of the strain *w*CfeJ deposited in GenBank under accession no. CP116768. ^3^supergroup V assigned by Sharma & Som^116^. ^4^supergroup V assigned by Mioduchowska et al.^115^. ^5^supergroup W assigned by Beliavskaia et al.^117^. ^6^supergroup W assigned by Sharma & Som^116^.

The phylogeny of *Wolbachia* in *T*. *stenaspis* was based on sequence analysis of six bacterial genes: 16S rRNA, *coxA*, *fbpA*, *ftsZ*, *gatB* and *hcpA* (2523 bp in total). Both individual gene sequences, as well as the six-gene phylogenetic characterization indicated that bacteria from the spider formed a separate supergroup. Our research revealed that *Wolbachia* from *T*. *stenaspis* belongs to a new *Wolbachia* supergroup X (Fig. 3). We ruled out the possibility that the spider endosymbiont could be a recombinant between strains of other subgroups, as the φ test did not reveal statistically significant evidence for recombination (*p* = 1).

Phylogenetic trees reconstructing the relationships of bacteria based on the analyses of individual genes are shown in Figs. S1–S6 available in the online Supplementary Material. The phylogeny based on the concatenated set of 16S rRNA, *coxA*, *fbpA*, *ftsZ*, *gatB* and *hcpA* gene sequences is depicted in Fig. 3. An additional phylogenetic tree showing that *Wolbachia* from *T*. *stenaspis* does not group with *Wolbachia* supergroups discovered so far in spiders, i.e. supergroups A and B is presented in Fig. S9 available in the online Supplementary Material.

## Discussion

### Long-term spider survival rates

**Fig. 4:**
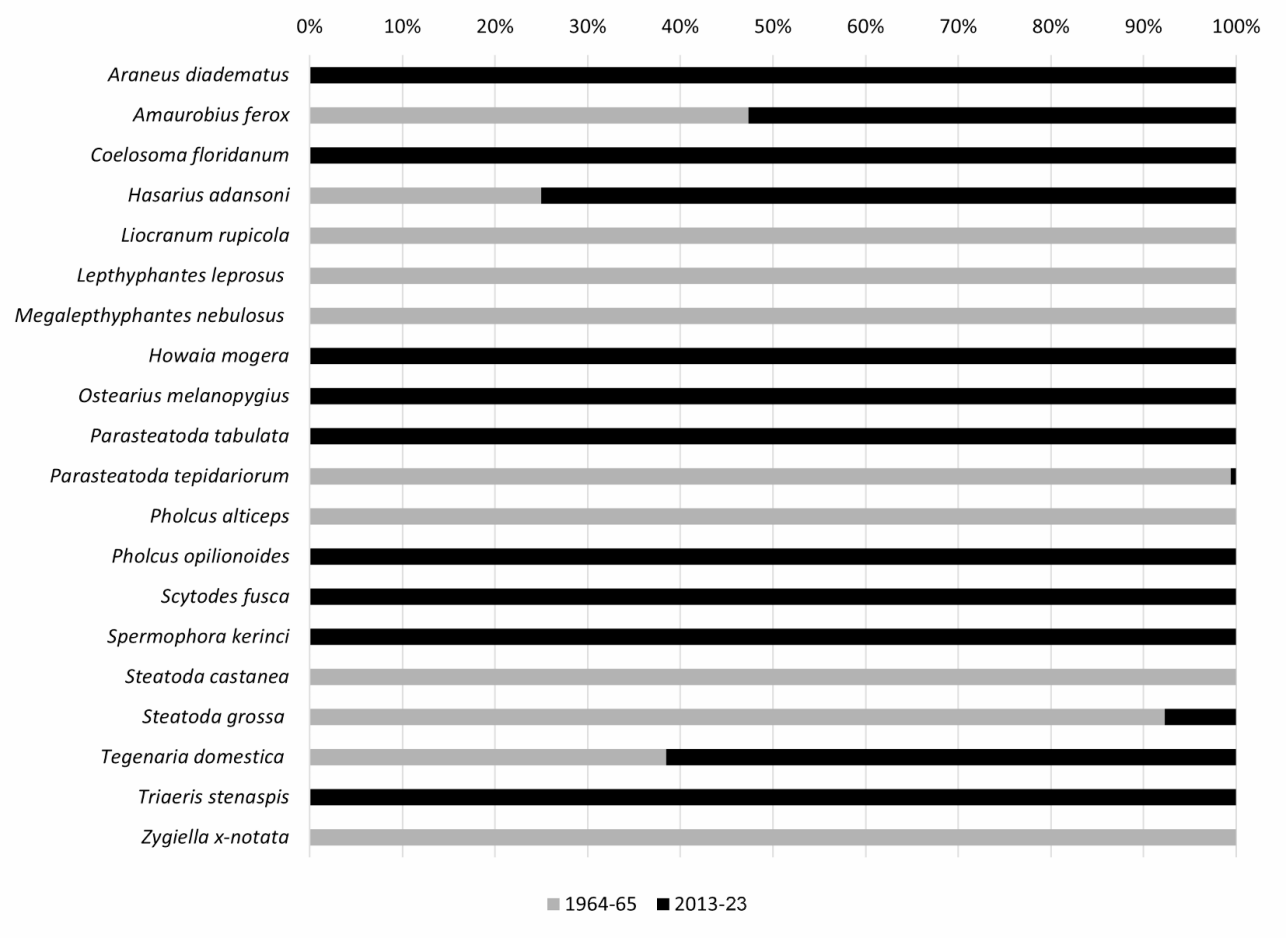
Percentages of spider species in the Poznań Palm House in 2013–2023 compared to unpublished data of Woźniczko^33^.

Presently, we compare the old findings originated from 1966 with the results of our studies carried out in 2013–2023. Woźniczko^33^ listed 11 spider species, of which 2 were designated as spiders of foreign origin: *H. adansoni* and *P. tepidariorum*, with the latter one being most numerous (77%). As a result of the current series of studies conducted after more than 50 years, 18 taxa have been identified, of which 14 were classified to the species level. The material acquired included 9 species of spiders characterized as allochthonous to the European fauna. Despite potential fluctuations in environmental conditions between the two distant study, attributed to factors such as the introduction of novel elements through horticultural plants (inclusive of substrate-dwelling invertebrates, bacteria and fungi), routine utility works, unpredictable variations in temperature and humidity, a restricted and simplified food base, and frequent application of plant protection pesticides, the persistence of 5 spider species has been confirmed between 1966 and the current survey cycle. Among them, 2 were alien species of spiders: *H. adansoni* and *P. tepidariorum*, and 3 were native species: *Amaurobius ferox* (Walckenaer, 1830), *Steatoda grossa* (C. L. Koch, 1838) and *Tegenaria domestica* (Clerck, 1758) (Fig. 4).

Based on these findings, we considered the aforementioned species a stable faunistic element of the Poznań Palm House. Although two of them are native to remote geographic zones (*H. adansoni*—African origin, *P. tepidariorum*—Asian origin), and despite frequent use of insecticides, which could limit spider abundance and their predation rate^81–85^, they have not caused lethal effects to these two species. Our suggestion posits that *H. adansoni* and *P. tepidariorum* may exhibit resistance to these chemical agents, which is consistent with the findings of Wilczek et al.^86^, Babczyńska et al.^87^ and Řezáč et al.^88^. These authors confirmed the relative resistance of certain spider species to chemical contamination and tolerance for high concentrations of heavy metals and some pesticides. All alien species recorded in the Poznań Palm House should be regarded as highly acclimated to demanding and fluctuating conditions, notably distinct from their natural habitats. This adaptability is especially important for spiders from tropical regions, as their occurrence has been confirmed in similar places like palm houses, greenhouses (butterfly, orchid etc.) and botanical gardens across Europe^7,14,15,18,19,62,64,65,89–91,,71,92–96^.

### Risk of colonization of synanthropic and natural habitats by alien species

The likelihood of alien spider species invading natural habitats located outside urban tropical island appears to be low^3^. This is due to the prevailing unfavorable thermal and humidity conditions of such habitats and highly competitive pressure of native species. Nevertheless, some species of non-native spiders, demonstrating broad ecological adaptability, have successfully acclimated to synanthropic and natural biotopes in Central Europe^97^. In the present study, we have found *O. melanopygius*, which inhabits natural and seminatural habitats in Poland and continues to expand its range^66,98–100^. It was originally described in New Zealand by Pickard-Cambridge^101^. This species successfully disseminated in Europe in the 20th century and has also recently spread rapidly in Poland^66,89^. The probable introduction of *O. melanopygius* into the palm house occurred through the transport of non native horticultural plants or substrates; however, it cannot be excluded that it could have entered the palm house with local plants and other materials delivered to the pavilion with temperate zone vegetation. An alternate scenario for its settlement was presented by Rozwałka & Stachowicz^100^. The latter authors suggested that *O. melanopygius* initially spread in the eastern part of Poland, primarily inhabiting greenhouses of horticultural farms and expanded to neighboring biotopes after the population stabilized. Passive dispersal by ballooning is an advantageous phenomenon that may allow the species to spread rapidly and colonize synanthropic and natural biotopes^66,89,98^. Another alien species, *H. mogera*, originated from Asian and was recorded for the first time in Europe in 2009^7^. In Poland, it has been identified in locations such as a botanical garden, garden store and butterfly house in zoological garden, where stable and numerous populations have been observed^60,66,102^. Considering that Jung et al.^103^ have found that this species inhabits stream banks with ruderal vegetation in agricultural, residential and industrial areas in South Korea, it is quite possible that this spider can also colonize seminatural habitats in Europe. Some species, due to their adaptation to environments heavily transformed by humans, are able to more easily colonize new synanthropic habitats. Two such examples are *P. tabulata* and *P. tepidariorum*, with *P. tabulata* being significantly more expansive^104^ and showing higher adaptability to natural biotopes^105^. *P. tabulata* has been recorded in cities, inhabiting building walls and fences, but also occurring in synanthropic habitats like roadside trees or forest car parks. Moreover, *H. adansoni* and *T. stenaspis* have also been found outside the Palm House in Poland. Thus far, they have been recorded in garden centers, ornamental plant markets and greenhouses. *H. adansoni* was found to be particularly numerous in a horticultural farm^60,66^, while *T. stenaspis* in a greenhouse with orchids^60^.

Although negative impact of alien invaders on spider assemblages in natural habitats has not been observed in Europe^8,106,107^, such risks continue to increase due to climate change. There is a general consensus that global warming will potentially promote the spread, colonization and development of invasive alien species in terrestrial as well as subterranean (caves) habitats^108–110^. Given the progressive climate warming in Poland^111,112^, it is possible that the thermophilic alien species found in the Poznań Palm House could extend their range to open land in the near future, potentially becoming invasive in both disturbed and natural habitats. Considering the potential for the rapid spread of certain alien spider species in Europe, such as the highly invasive *Mermessus trilobatus* (Emerton, 1882)^113,114^, it is crucial to exercise special care and monitor the abundance of invaders living in urban ecological islands such as palm houses.

### Endosymbiotic *Wolbachia* in *Triaeris stenaspis*

The capacity for parthenogenesis and high ecological tolerance appear to be pivotal factors enabling some arachnids to colonize such ecological islands as tropical palm houses^39^. Zawierucha et al.^32^ reported the occurrence of parthenogenetic species of Schizomida (*Stenochrus portoricensis* Chamberlin, 1922) in the Poznań Palm House in the past. The ability of females to reproduce without a male can be induced by endosymbiotic bacteria, with *Wolbachia* being the most prevalent among them^40,41^. Considering the fact that this type of reproduction is advantageous for species to become successful colonizers^11^, we focused on the parthenogenetic alien spider *T. stenaspis*. Until now, the presence of endosymbiotic bacteria like *Wolbachia* or *Cardinium* in *T. stenaspis* has not been confirmed^36^. This report marks the first identification of *Wolbachia* in this species. The endosymbiont was characterized based on the sequences of six bacterial housekeeping genes: 16S rRNA, *coxA*, *fbpA*, *ftsZ*, *gatB* and *hcpA*. Sequence identity analysis of single genes revealed the highest complementarity to DNA of different *Wolbachia* supergroups from insect hosts representing various orders, including Isoptera, Hemiptera, Lepidoptera, Siphonaptera and Odonata. Specifically, the DNA sequences of the endosymbiont from *T*. *stenaspis* showed the highest identity with the genes of the following *Wolbachia* supergroups: B (*Wolbachia* from *A*. *betuletana* and *I*. *elegans*), I (*Wolbachia w*Cfe from *C*. *felis*), F (*Wolbachia* from *N*. *takasagoensis* and *C*. *echiniformis*), V (*Wolbachia w*CfeJ from *C*. *felis*), and additionally to an unassigned supergroup (*Wolbachia* from *N*. *muiri*). Our phylogenetic reconstruction of *Wolbachia* supergroups based on six loci revealed that the bacteria recovered from the spider clearly formed distinct *Wolbachia* lineages, differing from the known *Wolbachia* supergroups (Fig. 3). Furthermore, there is compelling evidence supporting the identification of a novel *Wolbachia* supergroup X from *T*. *stenaspis* characterized by unique 5’-TCATATC-3’ and 5’-GACTTCG-3’ sequences within the 16S rRNA and the *ftsZ* genes, respectively.

Supergroups V and W were duplicated due to the publication of independent supergroups with the same designation at the same time. Thus, there are currently two distinct supergroups V described in different groups of arthropods: Mioduchowska et al.^115^ discovered supergroup V in *Unio crassus* Philipsson, 1788 (Mollusca, Bivalvia) and *Streptocephalus cafer* Lovén, 1847 (Arthropoda, Branchipoda). Concurrently, Sharma & Som^116^ introduced a new *Wolbachia* supergroup V from *C. felis* (Arthropoda, Insecta). Similarly, Sharma & Som^116^ and Beliavskaia et al.^117^ designated two independent supergroups with the same name “supergroup W” from various host groups: *C*. *felis* and *Howardula* sp. (Nematoda, Secementea), respectively. It should be noted that although two independent supergroups V and W were detected in one host, i.e. *C*. *felis*, the *Wolbachia* strains were designated as *w*CfeJ (accession no. CP011157) and *w*CfeT (accession no. CP051156), respectively. In light of the duplication of supergroup names assigned to independent bacterial strains, it seems necessary to establish a committee to update the nomenclature of newly described *Wolbachia* supergroups. Researchers discovering a novel supergroup would be encouraged to submit relevant information to the committee to assign a logical designation for new supergroup.

The detection of *Wolbachia* in *T. stenaspis* leads us to propose a strong influence of this endosymbiont on the manipulation of the reproduction of this species towards parthenogenesis. This adaptation could potentially facilitate the colonization and establishment in the Poznań Palm House and similar “tropical islands” in Europe.

## Conclusions

(1) Thermophilic alien spider species are present in the Poznań Palm House. Given the progressive climate warming in Poland, it is possible that they could extend their range to open land in the near future, potentially becoming invasive in both disturbed and natural habitats. (2) We have detected the new *Wolbachia* supergroup X in *T. stenaspis*. The presence of the bacteria may be related to the induction of parthenogenesis in the spider as an adaptation for survival in isolated conditions.

## Electronic supplementary material

Below is the link to the electronic supplementary material.

Supplementary Material 1

Supplementary Material 2

Supplementary Material 3

Supplementary Material 4

Supplementary Material 5

Supplementary Material 6

Supplementary Material 7

Supplementary Material 8

Supplementary Material 9

Supplementary Material 10

Supplementary Material 11

Supplementary Material 12

Supplementary Material 13

Supplementary Material 14

## Data Availability

Sequencing data generated and analyzed in this study are deposited to NCBI Nucleotide Database (accession nos. OR456447, OR457752, OR450018, and OR462179–OR462182).
