# Supplementary figures and images for: Alien spiders in a palm house with the first report of parthenogenetic Triaeris stenaspis (Araneae: Oonopidae) infected by Wolbachia from new supergroup X
Source: Sci Rep. 2025 Mar 19;15:9512. doi: 10.1038/s41598-025-93540-1 (PMC11923183; doi:10.1038/s41598-025-93540-1)

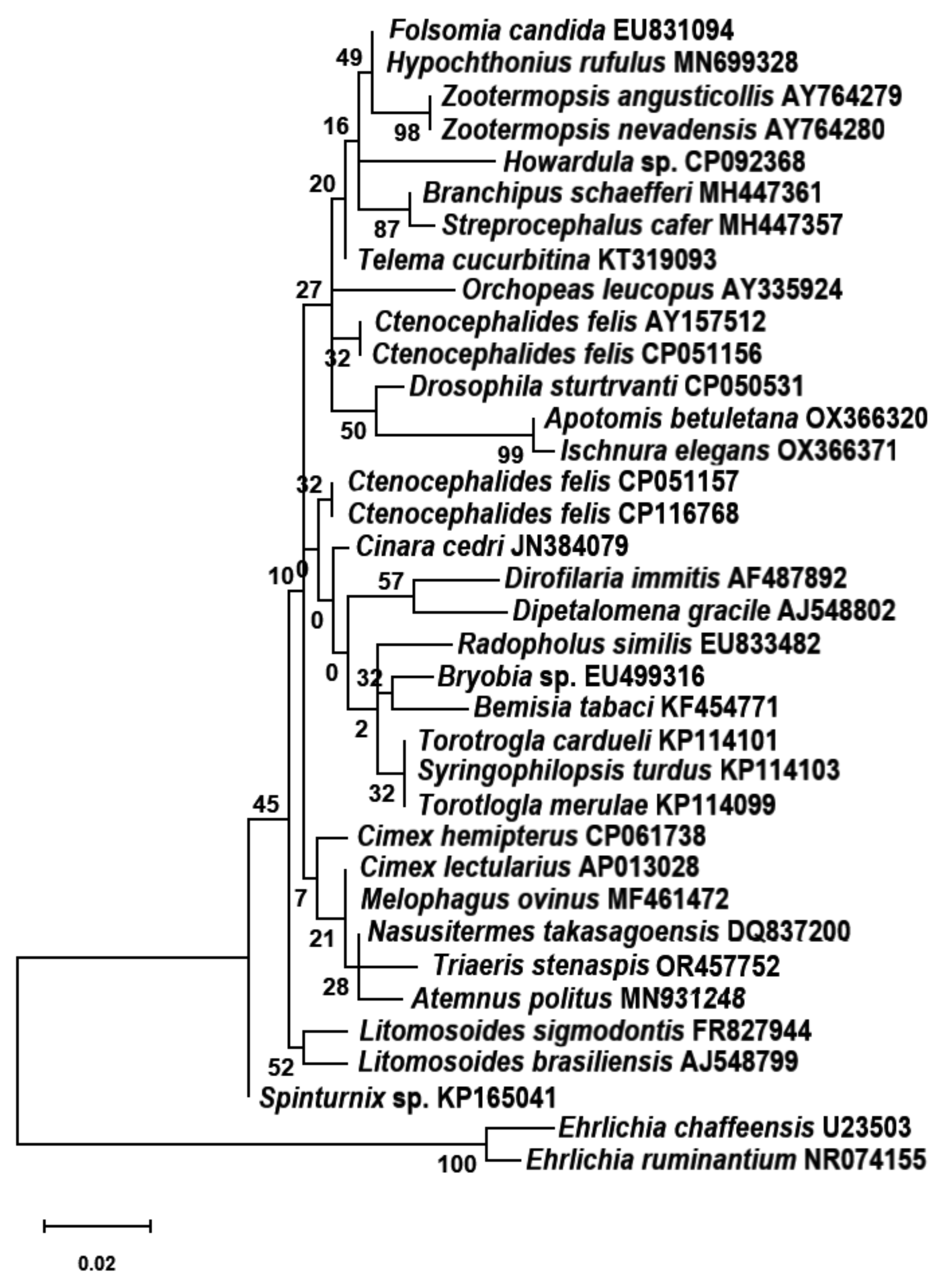

Supplement: Supplementary file 1 — Supplementary Material 1 [file 41598_2025_93540_MOESM1_ESM.tif]

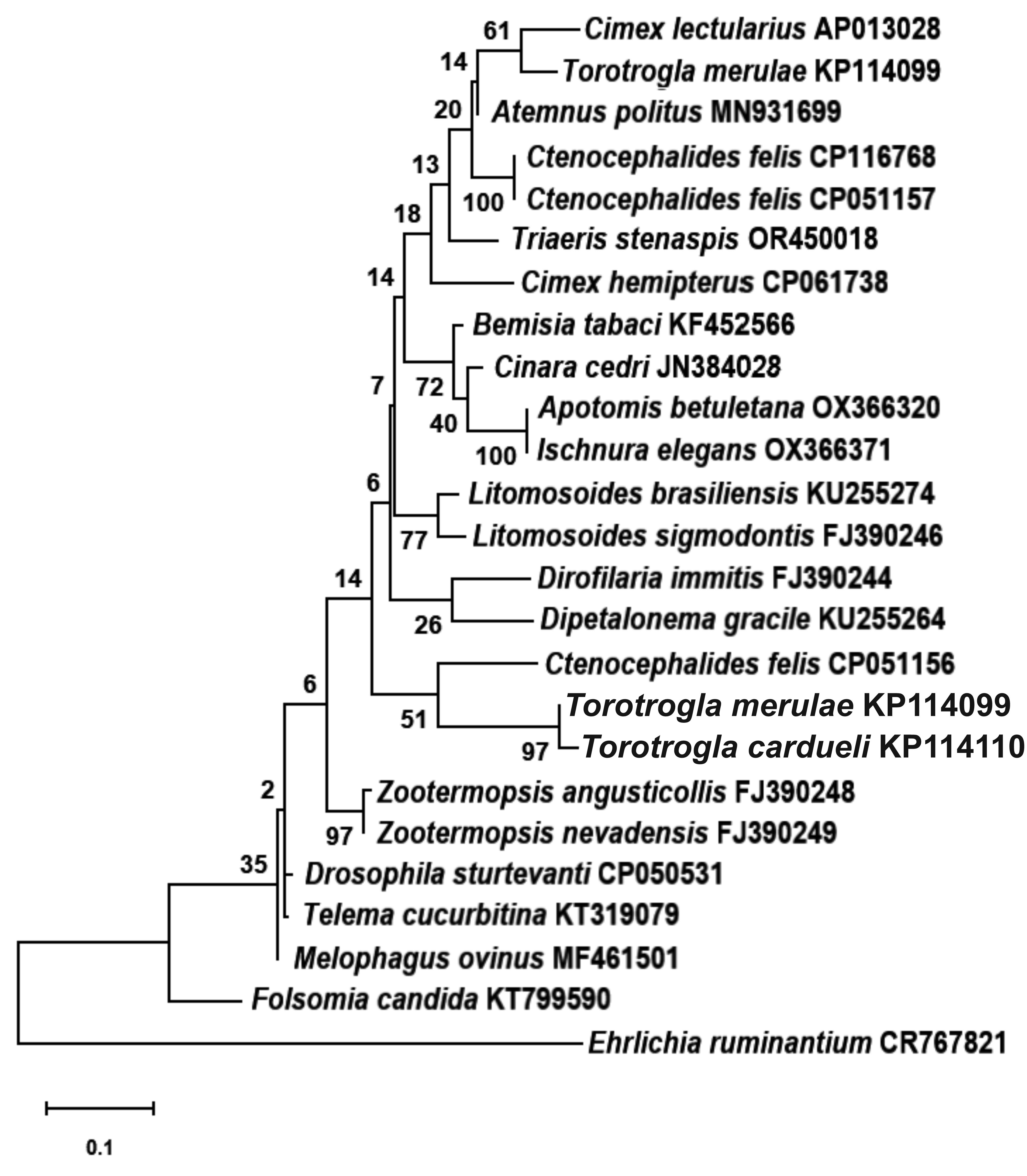

Supplement: Supplementary file 2 — Supplementary Material 2 [file 41598_2025_93540_MOESM2_ESM.tif]

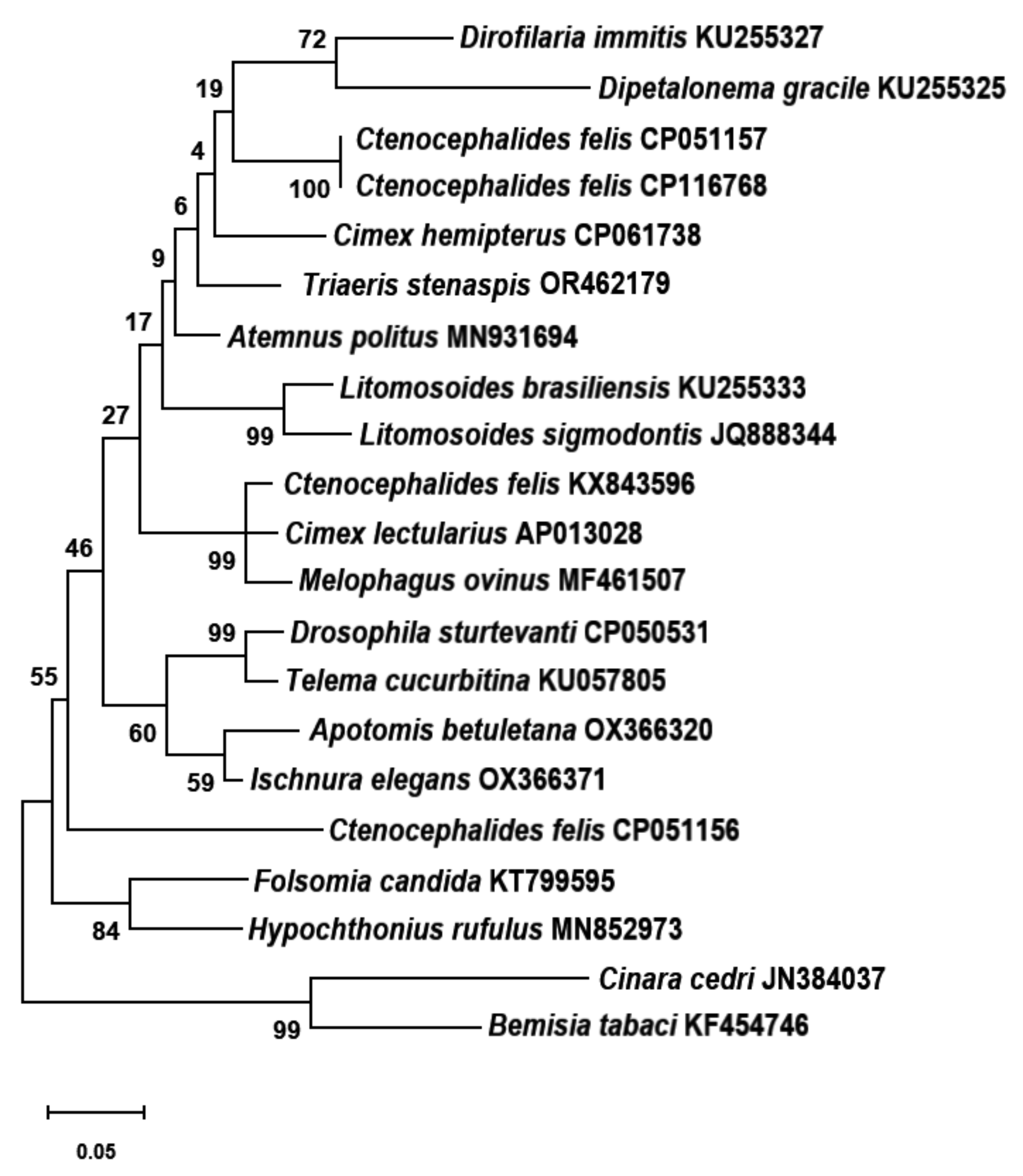

Supplement: Supplementary file 3 — Supplementary Material 3 [file 41598_2025_93540_MOESM3_ESM.tif]

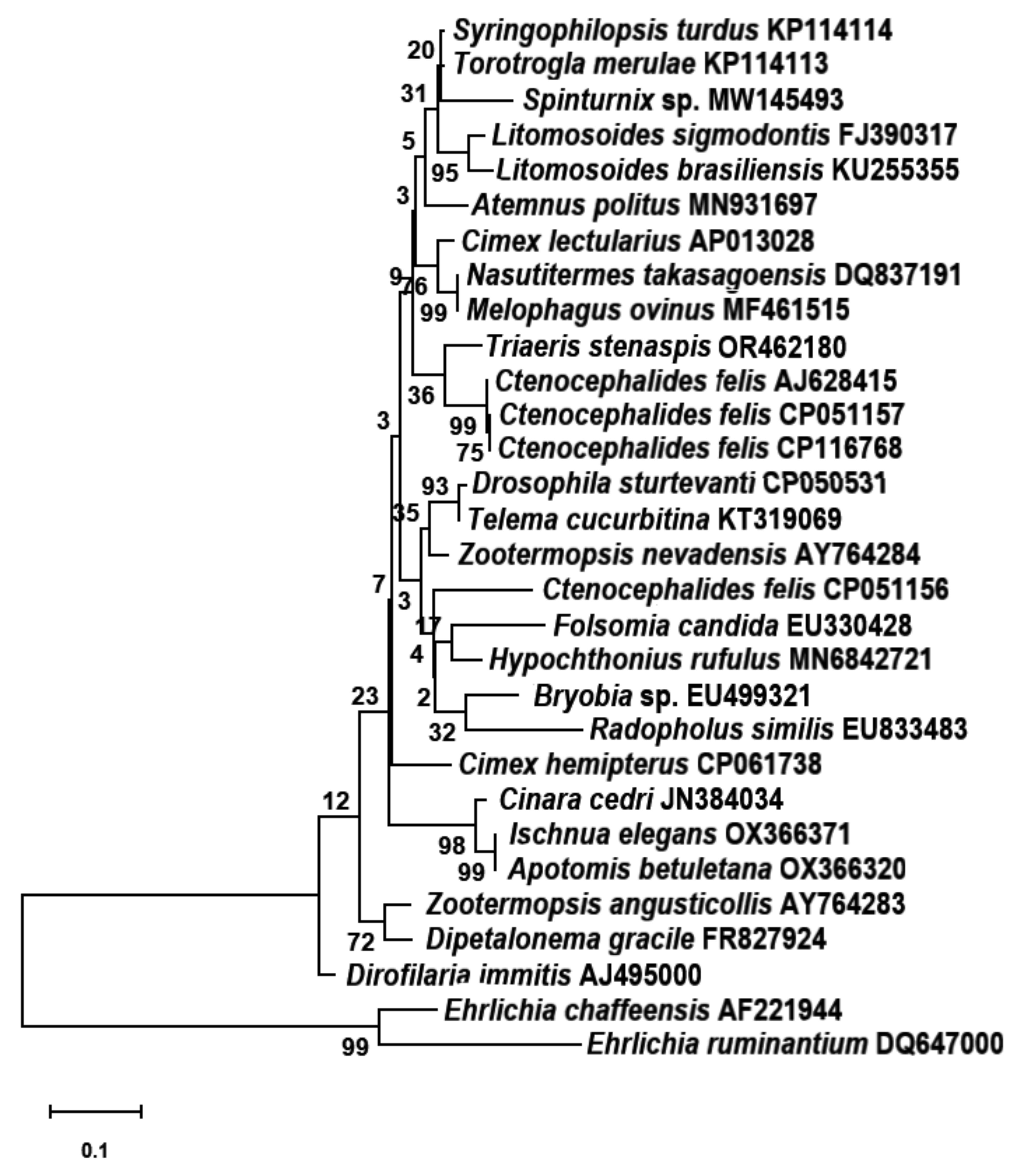

Supplement: Supplementary file 4 — Supplementary Material 4 [file 41598_2025_93540_MOESM4_ESM.tif]

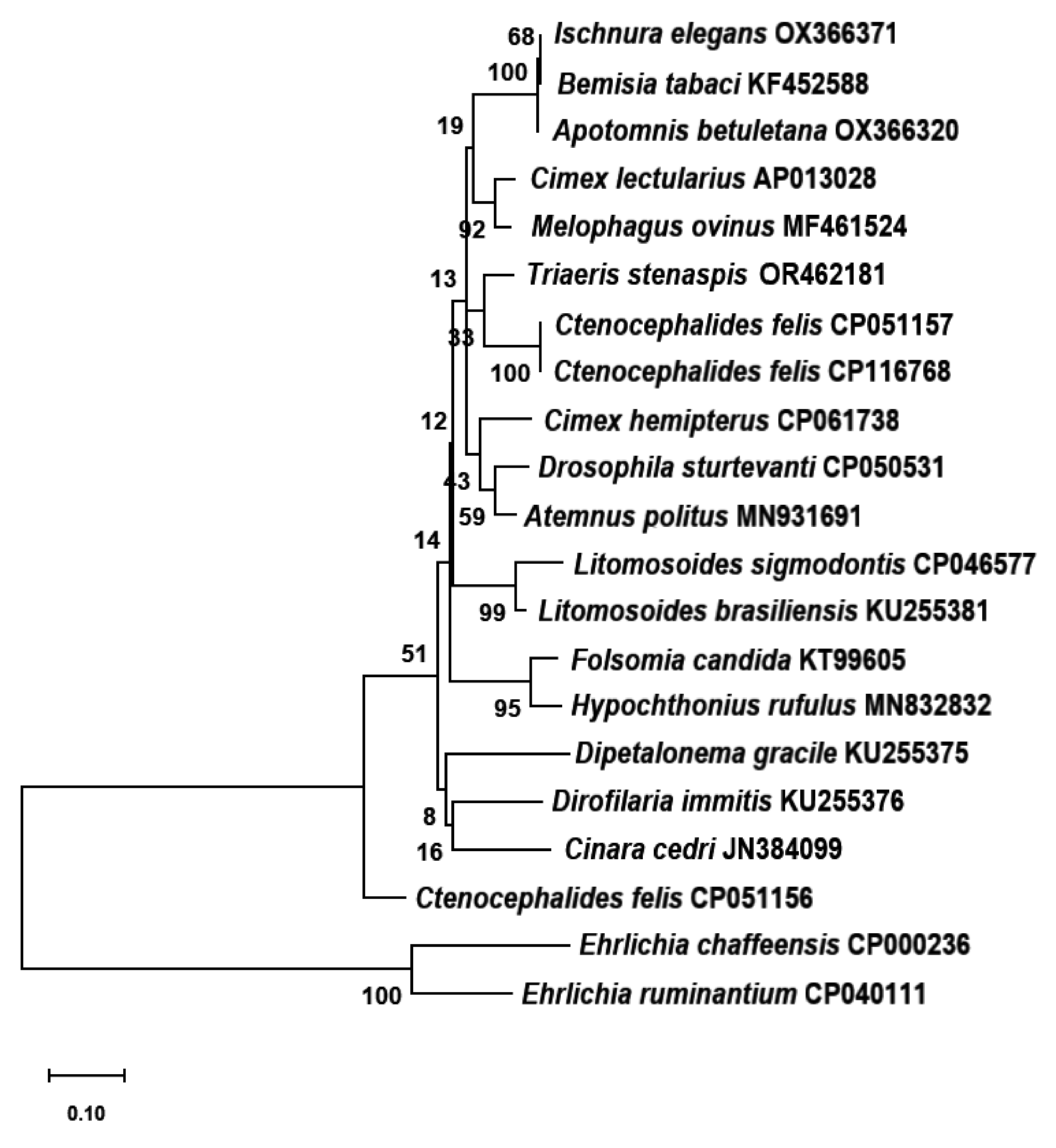

Supplement: Supplementary file 5 — Supplementary Material 5 [file 41598_2025_93540_MOESM5_ESM.tif]

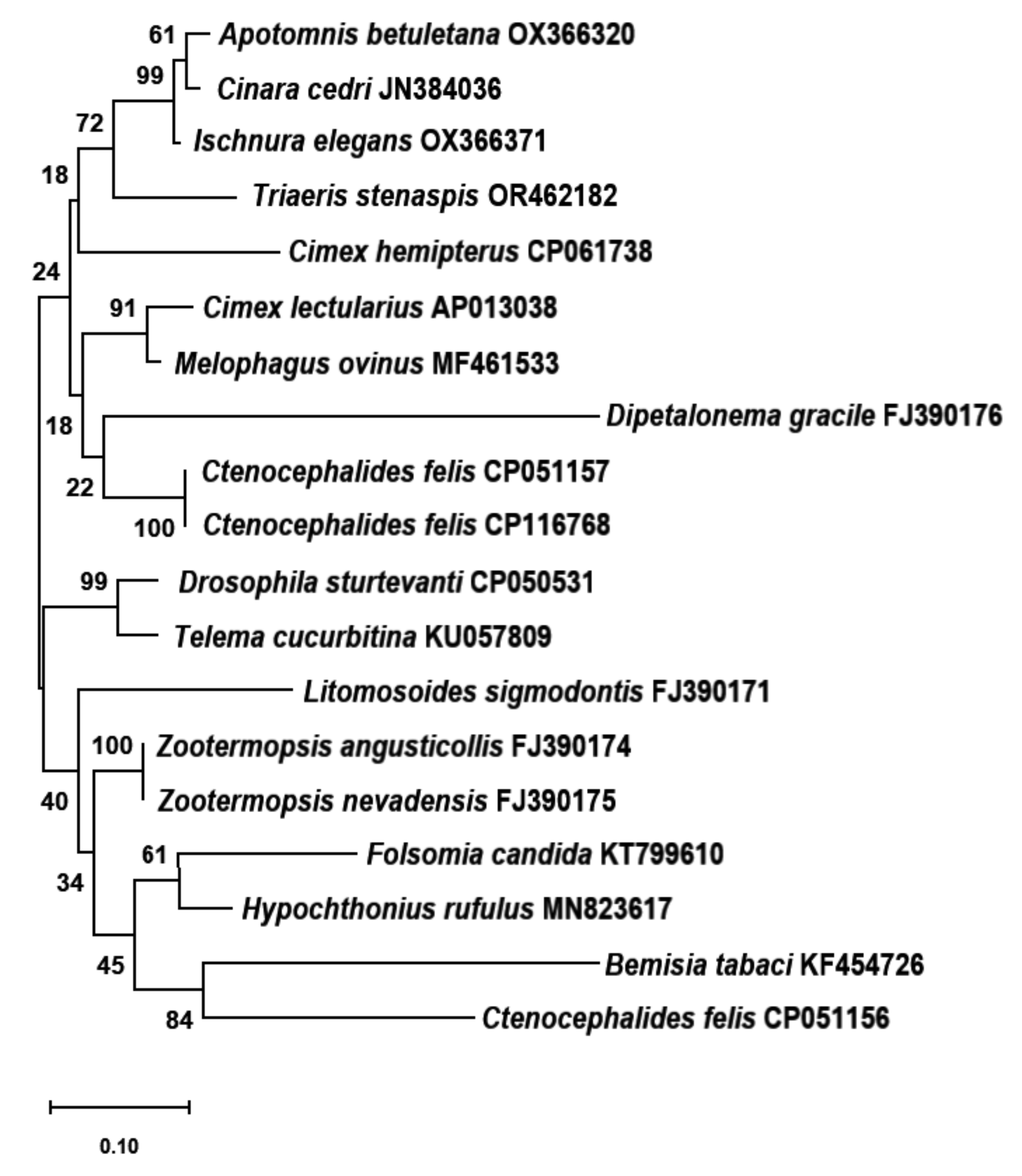

Supplement: Supplementary file 6 — Supplementary Material 6 [file 41598_2025_93540_MOESM6_ESM.tif]

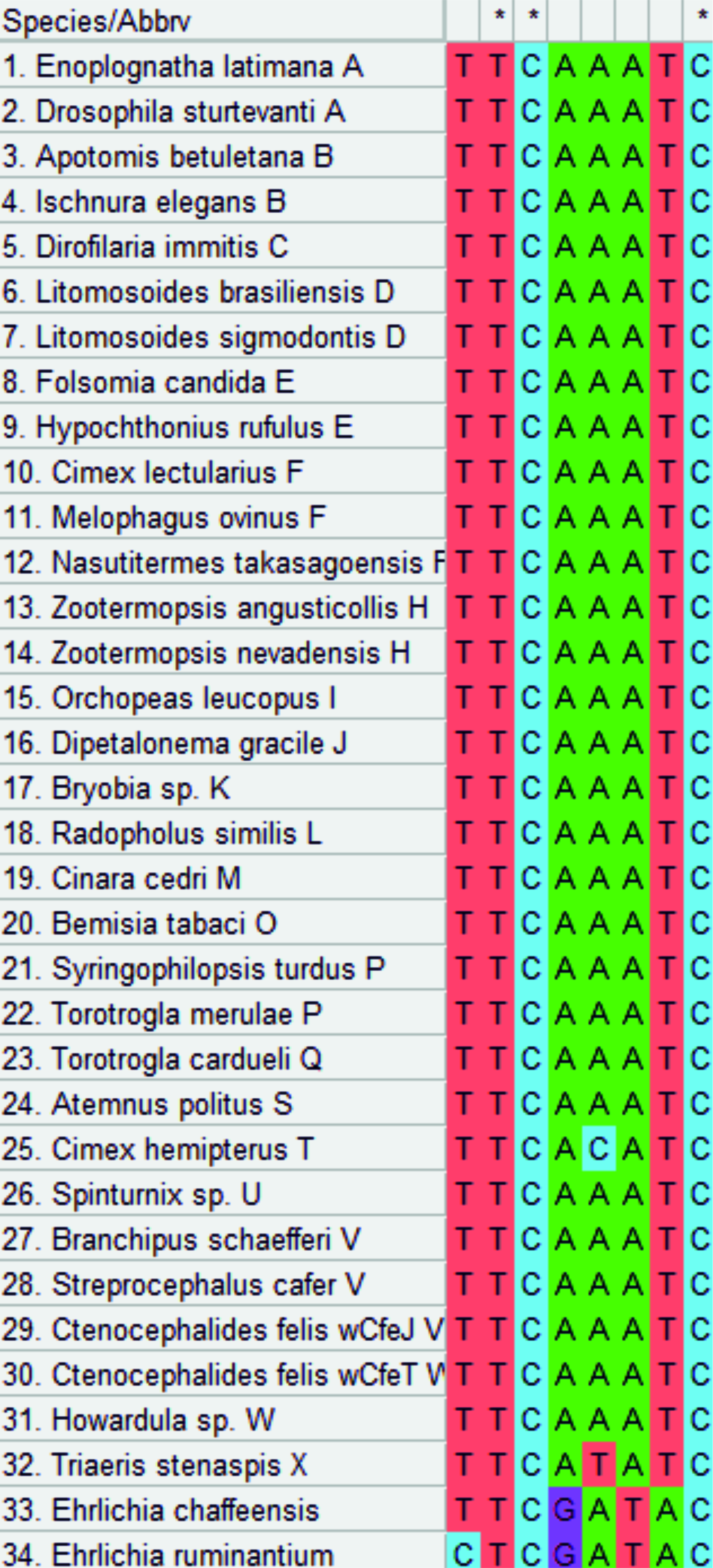

Supplement: Supplementary file 7 — Supplementary Material 7 [file 41598_2025_93540_MOESM7_ESM.tif]

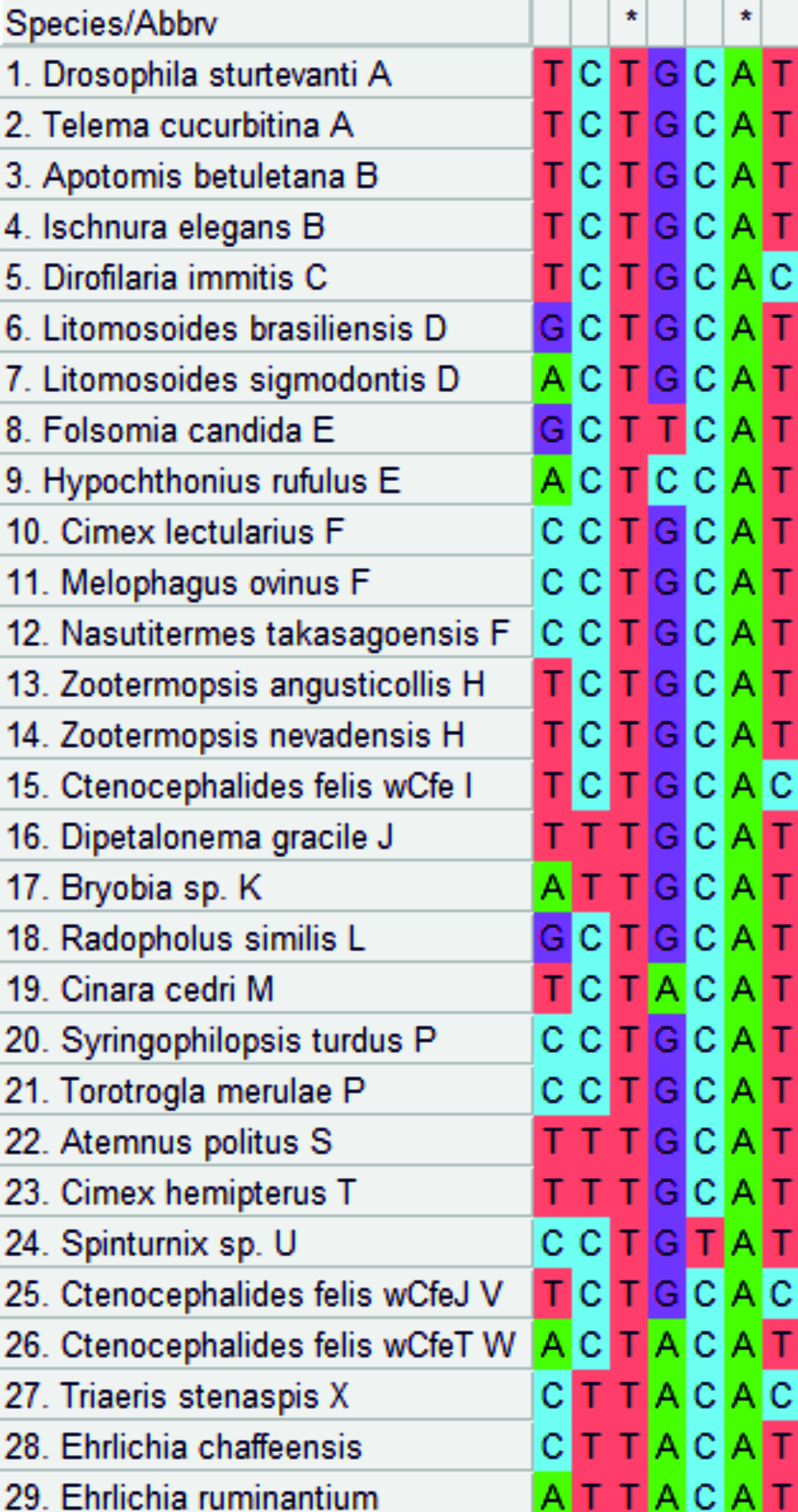

Supplement: Supplementary file 8 — Supplementary Material 8 [file 41598_2025_93540_MOESM8_ESM.tif]

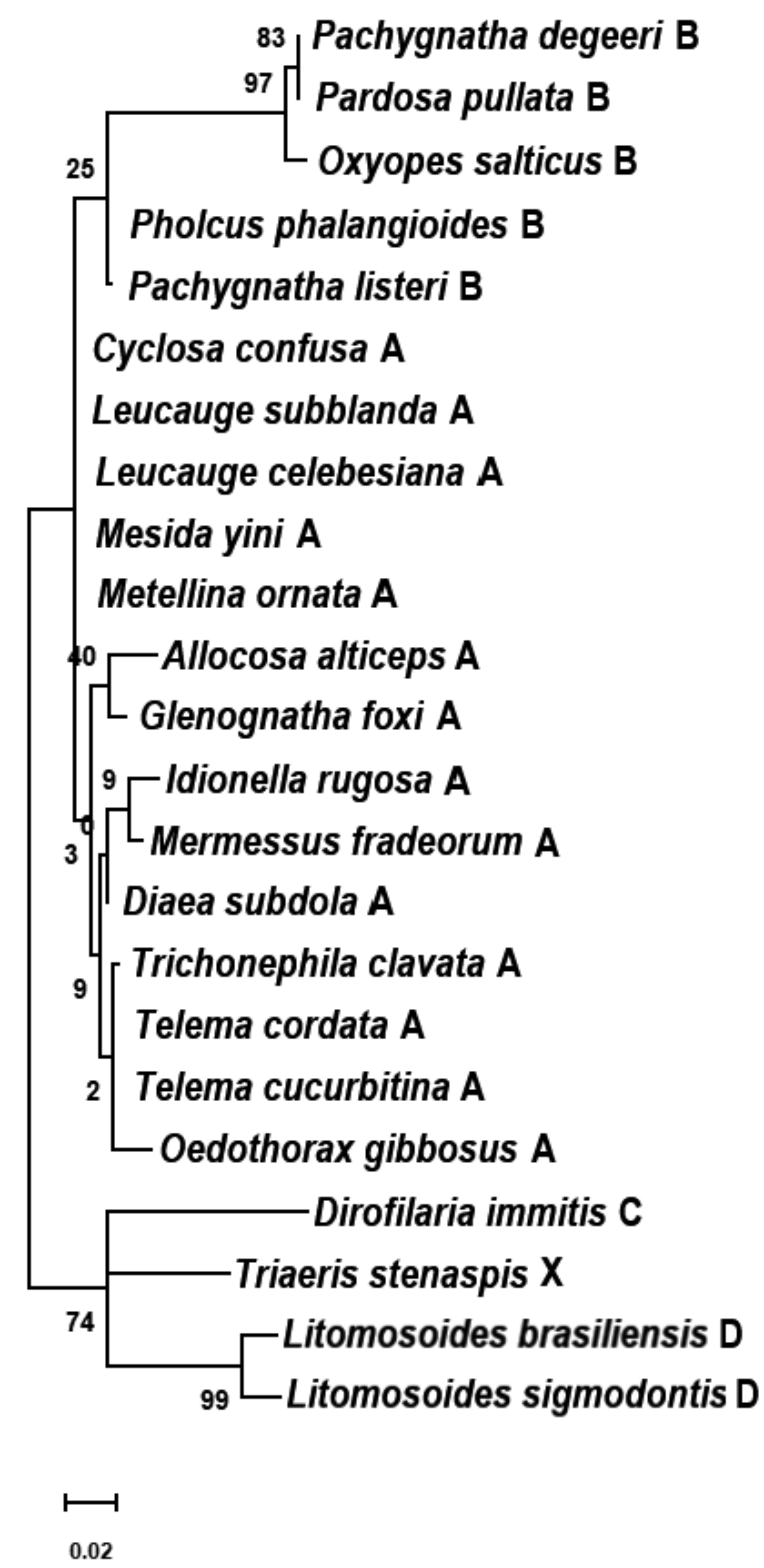

Supplement: Supplementary file 9 — Supplementary Material 9 [file 41598_2025_93540_MOESM9_ESM.tif]
