# Supplementary material for: Alien spiders in a palm house with the first report of parthenogenetic Triaeris stenaspis (Araneae: Oonopidae) infected by Wolbachia from new supergroup X
Source: Sci Rep. 2025 Mar 19;15:9512. doi: 10.1038/s41598-025-93540-1 (PMC11923183; doi:10.1038/s41598-025-93540-1)
Supplement: Supplementary file 10 — Supplementary Material 10 [file 41598_2025_93540_MOESM10_ESM.docx]

**Table S1.** Additional data on sampled material from Poznań Palm House. I-IX – pavilions: I – subtropical vegetation, II – temperate vegetation, V and VI – tropical vegetation, VII – aquatic vegetation, IX –xerophytes and savanna vegetation of old and new world, B – breeding room, C – underground corridor, S – storage room; Collectors: PS – Paweł Symkowiak, SBB – members of Invertebrate Research Group of Naturalist Science Club in Adam Mickiewicz University, SK – Szymon Konwerski, TR – Tomasz Rutkowski; * – alien species

| Species | Pavilon | Date | Collector |
| --- | --- | --- | --- |
| *Amaurobius ferox* | II, C | 25.11.2013, 02.11, 14.12.2015 | PS, SBB, TR |
| *Amaurobius* sp. | I | 21.02.2014, 20.04.2015 | TR |
| *Araneus diadematus* | B | 20.04.2015 | TR |
| *Coleosoma floridanum ** | I, VI, IX, C | 29.10, 25.11.2013, 17.11.2014, 02.03, 20.04, 02.11.2015 | PS, TR |
| *Dysdera* sp. | I | 17.11.2014 | PS |
| *Hasarius adansoni ** | I, VI, VII | 25.11, 29.10.2013, 17.11.2014, 20.04.2015 | PS, TR |
| *Howaia mogera ** | I, V, VI, C | 29.10.2013, 02.03, 20.04, 17.11, 14.12.2015, 06.04.2016 | PS, SBB, SK, TR |
| *Ostearius melanopygius ** | I | 29.10, 25.11.2013, 17.11.2014, 06.04.2016 | PS, SK |
| *Parasteatoda tabulata ** | I, V | 17.11.2014 | PS, TR |
| *Parasteatoda tepidariorum ** | C | 14.12.2015 | PS, SBB, TR |
| *Pholcus opilionoides* | C | 02.11.2015, 14.12.2015 | PS, SBB |
| *Pirata* sp. | S | 17.11.2014 | PS |
| *Scytodes fusca ** | I, C | 29.10.2013, 17.11.2014, 02.03.2015, 20.04.2015, 02.11.2015 | PS, TR |
| *Spermophora kerinci ** | V, VI | 02.03.2015, 20.04.2015 | PS, TR |
| *Steatoda grossa* | C | 14.12.2015 | PS, SBB |
| *Tegenaria domestica* | C | 02.11.2015, 14.12.2015 | PS, SBB |
| *Tegenaria* sp. | I | 20.04.2015 | TR |
| *Triaeris stenaspis ** | I | 17.11.2014, 17.04.2023 | PS |
