# Supplementary material for: Alien spiders in a palm house with the first report of parthenogenetic Triaeris stenaspis (Araneae: Oonopidae) infected by Wolbachia from new supergroup X
Source: Sci Rep. 2025 Mar 19;15:9512. doi: 10.1038/s41598-025-93540-1 (PMC11923183; doi:10.1038/s41598-025-93540-1)
Supplement: Supplementary file 11 — Supplementary Material 11 [file 41598_2025_93540_MOESM11_ESM.docx]

**Table S2.** Primer sets used for characterization of *Wolbachia* in the spider *Triaeris stenaspis*

| Primer designation | Primer sequence  (5’ → 3’) | Gene | Product | Annealing  temperature (ºC) | Reference |
| --- | --- | --- | --- | --- | --- |
| 553F_W  EHR16SR | CTTCATRYACTCGAGTTGCWGAGT  TAGCACTCATCGTTTACAGC | 16S rRNA | 16S rRNA | 55 | [69, 71] |
| gatB_F1  gatB_R1 | GAKTTAAAYCGYGCAGGBGTT  TGGYAAYTCRGGYAAAGATGA | *gatB* | glutamyltRNA(Gln) amidotransferase | 54 | [47] |
| hcpA_F1  hcpA_R1 | GAAATARCAGTTGCTGCAAA  GAAAGTYRAGCAAGYTCTG | *hcpA* | conserved hypothetical protein | 54 | [47] |
| fbpA_F1  fbpA_R1 | GCTGCTCCRCTTGGYWTGAT  CCRCCAGARAAAAYYACTATTC | *fbpA* | fructose-bisphosphate aldolase | 59 | [47] |
| coxA_F1  coxA_R1 | TTGGRGCRATYAACTTTATAG  CTAAAGACTTTKACRCCAGT | *coxA* | cytochrome c oxidase | 54 | [47] |
| ftsZ_F1  ftsZ_R1 | TTGCAGAGCTTGGACTTGAA  CATATCTCCGCCACCAGTAA | *ftsZ* | prokaryotic cell division protein | 55 | [72] |
