# Supplementary material for: Alien spiders in a palm house with the first report of parthenogenetic Triaeris stenaspis (Araneae: Oonopidae) infected by Wolbachia from new supergroup X
Source: Sci Rep. 2025 Mar 19;15:9512. doi: 10.1038/s41598-025-93540-1 (PMC11923183; doi:10.1038/s41598-025-93540-1)
Supplement: Supplementary file 12 — Supplementary Material 12 [file 41598_2025_93540_MOESM12_ESM.docx]

**Table S3.** GenBank accession numbers of sequence data used in this study to align *Wolbachia* from *Triaeris stenaspis*

| *Wolbachia* host | *Wolbachia* phylogenetic group | GenBank accession no. | | | |  |  |
| --- | --- | --- | --- | --- | --- | --- | --- |
|  |  | 16S rRNA | *gatB* | *ftsZ* | *hcpA* | *coxA* | *fbpA* |
| *Drosophila sturtevanti* | A | CP050531 | CP050531 | CP050531 | CP050531 | CP050531 | CP050531 |
| *Telema cucurbitina* | A | KT319093 |  | KT3119069 | KU057809 | KT319079 | KU057805 |
| *Apotomis betuletana* | B | OX366320 | OX366320 | OX366320 | OX366320 | OX366320 | OX366320 |
| *Ischnura elegans* | B | OX366371 | OX366371 | OX366371 | OX366371 | OX366371 | OX366371 |
| *Dirofilaria immitis* | C | AF487892 | KU255376 | AJ495000 |  | FJ390244 | KU255327 |
| *Litomosoides sigmodontis* | D | FR827944 | CP046577 | FJ390317 | FJ390171 | FJ390246 | JQ888344 |
| *Litomosoides brasiliensis* | D | AJ548799 | KU255381 | KU255355 |  | KU255274 | KU255333 |
| *Folsomia candida* | E | EU831094 | KT99605 | EU330428 | KT799610 | KT799590 | KT799595 |
| *Hypochthonius rufulus* | E | MN699328 | MN832832 | MN6842721 | MN823617 |  | MN852973 |
| *Nasutitermes takasagoensis* | F | DQ837200 |  | DQ837191 |  |  |  |
| *Melophagus ovinus* | F | MF461472 | MF461524 | MF461515 | MF461533 | MF461501 | MF461507 |
| *Cimex lectularius* | F | CP061738 | AP013028 | AP013028 | AP013028 | AP013028 | AP013028 |
| *Zootermopsis angusticollis* | H | AY764279 |  | AY764283 | FJ390174 | FJ390248 |  |
| *Zootermopsis nevadensis* | H | AY764280 |  | AY764284 | FJ390175 | FJ390249 |  |
| *Ctenocephalides felis w*Cfe | I | AY157512 |  | AJ628415 |  |  | KX843596 |
| *Orchopeas leucopus* | I | AY335924 |  |  |  |  |  |
| *Dipetalonema gracile* | J | AJ548802 | KU255375 | FR827924 | FJ3990176 | KU255264 | KU255325 |
| *Bryobia* sp. | K | EU499316 |  | EU499321 |  |  |  |
| *Radopholus similis* | L | EU833482 |  | EU833483 |  |  |  |
| *Cinara cedri* | M | JN384079 | JN384099 |  | JN384036 | JN384028 | JN384037 |
| *Bemisia tabaci* | O | KF454771 | KF452588 |  | KF454726 | KF452566 | KF454746 |
| *Syringophilopsis turdus* | P | KP114103 |  | KP114114 |  |  |  |
| *Torotrogla merulae* | P | KP114099 |  | KP114113 |  | KP114099 |  |
| *Torotrogla cardueli* | Q | KP114101 |  |  |  | KP114110 |  |
| *Atemnus politus* | S | MN931248 | MN931691 | MN931697 |  | MN931699 | MN931694 |
| *Cimex hemipterus* | T | CP061738 | CP061738 | CP061738 | CP061738 | CP061738 | CP061738 |
| *Spinturnix* sp. | U | KP165041 |  | MW145493 |  |  |  |
| *Branchipus schaefferi* | V | MH447361 |  |  |  |  |  |
| *Streprocephalus cafer* | V | MH447357 |  |  |  |  |  |
| *Ctenocephalides felis w*CfeJ | V | CP051157 | CP051157 | CP051157 | CP051157 | CP051157 | CP051157 |
| *Ctenocephalides felis w*CfeJ | V | CP116768 | CP116768 | CP116768 | CP116768 | CP116768 | CP116768 |
| *Howardula* sp. | W | CP092368 |  |  |  |  |  |
| *Ctenocephalides felis w*CfeT | W | CP051156 | CP051156 | CP051156 | CP051156 | CP051156 | CP051156 |
| *Triaeris stenaspis* | X | OR457752 | OR462181 | OR462180 | OR462182 | OR450018 | OR462179 |
|  |  |  |  |  |  |  |  |
| Outgroup strains |  |  |  |  |  |  |  |
| *Ehrlichia chaffeensis* |  | U23503 | CP000236 | AF221944 |  |  |  |
| *Ehrlichia ruminantium* |  | NR074155 | CP040111 | DQ647000 |  | CR767821 |  |
