# Supplementary material for: Alien spiders in a palm house with the first report of parthenogenetic Triaeris stenaspis (Araneae: Oonopidae) infected by Wolbachia from new supergroup X
Source: Sci Rep. 2025 Mar 19;15:9512. doi: 10.1038/s41598-025-93540-1 (PMC11923183; doi:10.1038/s41598-025-93540-1)
Supplement: Supplementary file 13 — Supplementary Material 13 [file 41598_2025_93540_MOESM13_ESM.docx]

**Table S4.** GenBank accession numbers of aligned *Wolbachia* sequences derived from spiders

| *Wolbachia* spider host | *Wolbachia* supergroup | GenBank accession no. | | | | | |
| --- | --- | --- | --- | --- | --- | --- | --- |
|  |  | 16S rRNA | *coxA* | *fbpA* | *ftsZ* | *gatB* | *hcpA* |
| *Allocosa alticeps* | A | KU041135 |  |  |  |  |  |
| *Cyclosa confusa* | A |  | KX169181 | MN202127 | KX380701 | MN202026 | MN202067 |
| *Diaea subdola* | A |  | MN202048 |  | MN202108 | MN202027 | MN202069 |
| *Glenognatha foxi* | A | MK529774 |  | MK631936 |  |  |  |
| *Idionella rugosa* | A | MK529770 | MK631922 | MK631923 | MK631924 | MK631925 | MK631926 |
| *Leucauge celebesiana* | A |  | KX169177 | MN202130 | KX380698 | MN202031 | MN202072 |
| *Leucauge subblanda* | A |  | KX169173 |  | MN202113 |  | MN202073 |
| *Mesida yini* | A |  | KX169178 | MN202132 | KX380706 | MN202033 | MN202075 |
| *Metellina ornata* | A |  | MN202053 | MN202131 | KX380693 | MN202032 | MN202074 |
| *Oedothorax gibbosus* | A | OW370537 | OW370537 | OW370567 | OW370567 | OW370537 | OW370567 |
| *Pardosa laura* | A |  | MN202060 |  | MN202121 | MN202039 |  |
| *Pinelema cordata* | A | KT319086 | KT319078 |  | KT319070 |  |  |
| *Pinelema cucurbitina* | A | KT319093 | KT319085 | KU057806 | KT319069 |  | KU057809 |
| *Trichonephila clavata* | A | AF232234 |  | MN202136 | AF232235 | MN202038 | MN202079 |
| *Alopecosa pulverulenta* | B | EU333932 |  |  |  |  |  |
| *Oxyopes salticus* | B | MK529773 |  |  |  |  |  |
| *Pachygnatha degeeri* | B | EU333936 |  |  |  |  |  |
| *Pardosa mionebulosa* | B |  |  | MN202137 | MN202122 | MN202040 | MN202080 |
| *Pardosa pullata* | B | EU333933 |  |  |  |  |  |
| *Philodromus subaureolus* | B |  | MN202062 | MN202138 | MN202132 | MN202041 | MN202081 |
| *Triaeris stenaspis* | X | OR457752 | OR450018 | OR462179 | OR462180 | OR462181 | OR462182 |
|  |  |  |  |  |  |  |  |
| Comparative material from other hosts |  |  |  |  |  |  |  |
| *Dirofilarial immitis* |  | AF487892 | FJ390244 | KU255327 | AJ495000 | KU255376 |  |
| *Litomosoides sigmodontis* |  | FR827944 | FJ390246 | JQ888344 | FJ390317 | CP046577 | FJ390171 |
| *Litomosoides brasiliensis* |  | AJ548799 |  |  | KU255355 | KU255381 |  |
