# Supplementary material for: Alien spiders in a palm house with the first report of parthenogenetic Triaeris stenaspis (Araneae: Oonopidae) infected by Wolbachia from new supergroup X
Source: Sci Rep. 2025 Mar 19;15:9512. doi: 10.1038/s41598-025-93540-1 (PMC11923183; doi:10.1038/s41598-025-93540-1)
Supplement: Supplementary file 14 — Supplementary Material 14 [file 41598_2025_93540_MOESM14_ESM.pdf]

SUPPLEMENTARY INFORMATION

Supplementary Table S1. Additional data on the sampled material from the Poznań Palm House. I-IX – pavilions: I – subtropical vegetation, II – temperate vegetation, V and VI – tropical vegetation, VII – aquatic vegetation, IX – xerophytes and savanna vegetation of old and new world, B – breeding room, C – underground corridor, S – storage room; Collectors: PS – Paweł Szymkowiak, SBB – members of the Invertebrate Research Group of the Naturalist Science Club at Adam Mickiewicz University, SK – Szymon Konwerski, TR – Tomasz Rutkowski; * – alien species

Supplementary Table S2. Primer sets used for characterization of *Wolbachia* in the spider *Triaeris stenaspis*

Supplementary Table S3. GenBank accession numbers of sequence data used in this study to align *Wolbachia* from *Triaeris stenaspis*

Supplementary Table S4. GenBank accession numbers of aligned *Wolbachia* sequences derived from spiders

Supplementary Figure S1. Maximum likelihood reconstruction of *Wolbachia* supergroup phylogeny based on the 16S rRNA sequences using MEGA 11 software. Strains are denoted by their host names, except for outgroup bacteria. Bar, substitutions per nucleotide. Bootstrap values based on 1000 replicates are shown on branches.

Supplementary Figure S2. Maximum likelihood reconstruction of *Wolbachia* supergroup phylogeny based on the *coxA* gene sequences using MEGA 11 software. Strains are denoted by their host names, except for outgroup bacteria. Bar, substitutions per nucleotide. Bootstrap values based on 1000 replicates are shown on branches.

Supplementary Figure S3. Maximum likelihood reconstruction of *Wolbachia* supergroup phylogeny based on the *fbpA* gene sequences using MEGA 11 software. Strains are denoted

by their host names. Bar, substitutions per nucleotide. Bootstrap values based on 1000 replicates are shown on branches.

Supplementary Figure S4. Maximum likelihood reconstruction of *Wolbachia* supergroup phylogeny based on the *ftsZ* gene sequences using MEGA 11 software. Strains are denoted by their host names, except for outgroup bacteria. Bar, substitutions per nucleotide. Bootstrap values based on 1000 replicates are shown on branches.

Supplementary Figure S5. Maximum likelihood reconstruction of *Wolbachia* supergroup phylogeny based on the *gatB* gene sequences using MEGA 11 software. Strains are denoted by their host names, except for outgroup bacteria. Bar, substitutions per nucleotide. Bootstrap values based on 1000 replicates are shown on branches.

Supplementary Figure S6. Maximum likelihood reconstruction of *Wolbachia* supergroup phylogeny based on the *hcpA* gene sequences using MEGA 11 software. Strains are denoted by their host names. Bar, substitutions per nucleotide. Bootstrap values based on 1000 replicates are shown on branches.

Supplementary Figure S7. Alignment showing the unique 5'-TCATATC-3' sequence from the 16S rRNA gene of *Wolbachia* supergroup X from *Triaeris stenaspis*.

Supplementary Figure S8. Alignment showing the unique 5'-CTTACAC-3' sequence from the *ftsZ* gene of *Wolbachia* supergroup X from *Triaeris stenaspis*.

Supplementary Figure S9. Maximum likelihood reconstruction of *Wolbachia* supergroup (A, B, C and D form spider hosts) phylogeny based on concatenated sequence alignments of six bacterial loci (16S rRNA, *coxA*, *fbpA*, *ftsZ*, *gatB*, *hcpA*) using MEGA 11 software. Strains are denoted by their spider host names. Capital letters indicate individual *Wolbachia* supergroups. Bar, substitutions per nucleotide. Bootstrap values based on 1000 replicates are shown on branches.
